# Isolated primary intracranial myeloid sarcoma with neuromeningeal infiltration: A case report

**DOI:** 10.3892/ol.2015.2964

**Published:** 2015-02-13

**Authors:** JUN QIAN, QU CUI, YUANBO LIU, XIAOYAN LI, XUEFEI SUN, HONG ZHU, CHEN WANG

**Affiliations:** 1 Department of Hematology, Beijing Tiantan Hospital, Capital Medical University, Dongcheng, Beijing 100050, P.R. China; 2 Department of Internal Medicine, Beijing Tiantan Hospital, Capital Medical University, Dongcheng, Beijing 100050, P.R. China

**Keywords:** chemotherapy, myelodysplastic disorders, myeloid sarcoma

## Abstract

Myeloid sarcoma is a rare extramedullary malignant tumor, which is often accompanied by the development of systemic myeloid disease at various sites. The involvement of the central nervous system is uncommon and spinal cord compression is particularly rare. In November 2012, a 27-year-old male presented with a paroxysmal headache, accompanied by nausea and vomiting, which had persisted for one year, and eyesight deterioration that had been apparent for five months. Magnetic resonance imaging (MRI) indicated a space-occupying disorder, a craniotomy to resect the brain tumor was undertaken, the pathological diagnosis of which was myeloid sarcoma. Two months after receiving 40 Gy of radiotherapy, the patient experienced numbness of the right thigh. MRI of the lumbar spinal canal revealed a mass involved both inside and outside the lumbar spinal canal. Pathological examination of the mass following resection also indicated myeloid sarcoma. Immunohistochemical analysis was positive for the ETO fusion gene in the bone marrow. Following six cycles of chemotherapy treatment, the patient achieved complete remission. At present, the patient is stable and is attending follow-up examinations regularly.

## Introduction

Myeloid sarcoma is a malignant tumor consisting of myeloblasts, with or without maturation, at anatomical sites other than the bone marrow (1,2). Myeloid sarcoma may proceed, and in certain cases be concurrent with leukemic infiltration to the bone marrow, which leads to the blastic transformation of a myelodysplastic syndrome or a chronic myeloproliferative disorder (3–9). In total, ~19% of myeloid sarcomas invade the central nervous system via the bone marrow of the adjacent cranium, vertebrae or orbital bones (9–11). Although the primary emergence of intracranial myeloid sarcoma is rare, isolated primary intracranial myeloid sarcomas with neuromeningeal infiltration should be carefully considered. Currently, standard chemotherapeutical regimens are used to control myeloid sarcomas (9,12–16). The present study describes a case of intracranial myeloid sarcoma without primary evidence of hematological disorders, and discusses the associated diagnostic and therapeutic strategies. Written informed consent was obtained from the patient.

## Case report

In November 2012, a 27-year-old male was admitted to the Beijing Tiantan Hospital (Beijing, China) with a paroxysmal headache that had persisted for one year and numbness of the right thigh that had been apparent for two months. The patient had experienced occasional paroxysmal headaches accompanied by nausea and vomiting for one year. In the five months prior to admission, the patient’s eyesight had deteriorated. The patient also exhibited an unsteady gait due to the numbness of the right thigh. Magnetic resonance imaging (MRI) of the head revealed a space-occupying disorder beside the bilateral occipital sinus, which involved a posterior region of the superior sagittal sinus. The patient underwent a craniotomy for the resection of the brain tumor in April 2012. The tumor mass was grey/white in color, pliable and located in the bilateral occipital lobes. The mass exhibited clear borders and a rich blood supply, had adhered slightly to the surrounding brain tissues and had invaded the sagittal sinus, with evidence of tumor thrombosis. The surgery removed a 38×69×3-mm section of brain tissue. The post-surgical biopsy identified a myeloid sarcoma positive for myeloperoxidase (MPO), cluster of differentiation (CD)34 and Ki-67 (50%). Subsequently, the patient underwent routine post-operative radiation therapy (total dose, 40 Gy) for one month. In September 2012, the patient had suffered hypoesthesia and pains primarily on the right side in the anogenital region and along the anterior and inner femoral surfaces. The patient had also experienced difficulty in defecating. A lumbar MRI scan revealed a space-occupying lesion inside and outside of the lumbar spinal tube. In October 2012, a repeat MRI identified multiple space-occupying disorders in the lumbar spinal canal of L2–3 and S2–4. After three days, surgery was performed to resect the majority of the tumor mass, which subsequently relieved the pain in the right thigh. The post-operative biopsy confirmed a myeloid sarcoma. Further immunohistochemical analysis revealed that the neoplastic cells were positive for MPO, CD34, lysozyme, vimentin, CD99, CD56, CD68 and Ki-67 (20%), but negative for CD3, CD20, glial fibrillary acidic protein, CD79α and cytokeratin. A physical examination revealed that the superficial lymph nodes, liver and spleen were impalpable, but there was no tenderness of the sternum. Bilateral visual acuity was reduced and the lower visual field was contracted. Furthermore, the superficial sensations in the front and inside of the right thigh and perineal region were reduced. The morphological analysis, immunophenotyping and chromosome genotyping of the bone marrow were normal. The ETO fusion gene was positive, with a quantity of 0.44%. Myeloid-derived tumor cells, expressing CD117, CD34, CD33, CD56 and human leukocyte antigen-DR were detected in the cerebrospinal fluid and accounted for 1.49% of the total cellular components.

**Figure 1:**
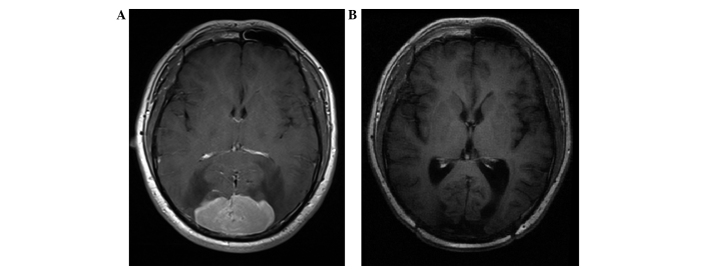
Magnetic resonance imaging revealing the intracranial myeloid sarcoma (A) prior to and (B) subsequent to the combined therapy.

**Figure 2:**
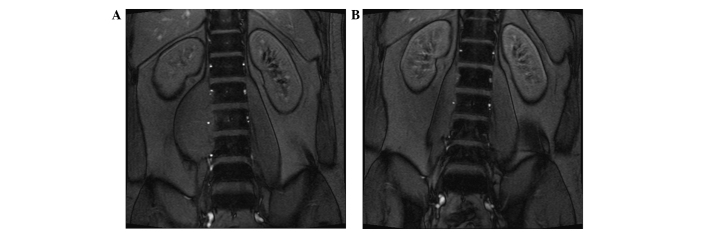
Magnetic resonance imaging of the lumbar myeloid sarcoma (A) prior to and (B) subsequent to the combined therapy.

Subsequent to the admission to the Beijing Tiantan Hospital in November 2012, the patient was administered chemotherapy in order to treat the myeloid sarcoma. The daily therapeutic regimen consisted of 10 mg idarubicin and 1.5 g cytarabine for three days, with intrathecal injections of 10 mg methotrexate and 5 mg dexamethasone. Following two rounds of chemotherapy, the patient’s eyesight returned to normal and the numbness of the right thigh was markedly relieved. Polymerase chain reaction amplification of the ETO fusion gene and immunophenotyping of the cerebrospinal were negative. MRI revealed that the spinal tumor mass had significantly decreased in size (Figs. 1 and 2). The patient was then transferred to a local hospital to receive a further four cycles of chemotherapy.

## Discussion

Myeloid sarcoma, which was previously known as granulocytic sarcoma or chloroma, is an uncommon malignant tumor characterized by the extramedullary blast proliferation of myeloid lineages that subsequently destroy the normal architecture of adjacent tissues (17). Three categories of myeloid sarcomas have been established as follows: i) Granulocytic sarcoma, which is the most common type; ii) monoblastic sarcoma, which is rare and primarily composed of monoblastic cells in patients with monoblastic leukemia; and iii) the rarer type of neoplasm that consists of three lineages of hematopoietic cells or blasts, and is usually associated with chronic bone marrow proliferative diseases (18). Myeloid sarcoma is able to invade any anatomical site, but affects the skin, gastrointestinal tract, lymph nodes and bones in particular. In addition, it is often found concurrently in patients with previously or recently recognized acute myeloid leukemia (AML), particularly monoblastic leukemia and AML with a t(8;21) translocation (17). Myeloid sarcoma may emerge prior to the appearance of blood or bone marrow disorders, and in such cases, should be considered synonymously alongside AML. Furthermore, myeloid sarcoma should be evaluated in terms of its morphological, phenotypical and genetic features for further classification into the AML subgroups (19).

Due to its low incidence, primary central nervous system myeloid sarcoma has not been widely reported. Myeloid sarcoma, as a group of heterogenetic diseases, presents with differential features depending upon the original affected site. Once a pathological diagnosis has been established, comprehensive evaluations, including bone marrow morphology, immunophenotyping, chromosomal banding and fusion gene analyses should be performed in order to determine an optimum treatment regimen (17). A study by Nishimura *et al* (17) revealed that AML patients with a t(8;21) translocation presented with extra-medullary sarcomas more frequently affecting the central nervous system. In the present study, the bone marrow morphology, and immunophenotyping and chromosomal banding examination results appeared normal, but the patient was positive for the presence of the ETO fusion gene. These results may be associated with the specificity and sensitivity of the methods that were used (20).

Due to the risk of leukemic cell metastasis to the spinal leptomeningeal tissues via the cerebrospinal fluid during the surgical excision of intraparenchymal myeloid sarcomas (19), a combined chemotherapy regimen, similar to one used for AML, is the recommended treatment for isolated intracranial myeloid sarcoma (9,19). As the central nervous system had been invaded by myeloid sarcoma cells in the patient of the present study, a regimen that combined intermediate doses of idarubicin and cytarabine was administered in order to facilitate the transport of the agents across the blood brain barrier (19,21). Due to the occurrence of post-chemotherapy grade IV myelosuppression, the dose of cytarabine could not be increased. Radiation therapy therefore provides an alternative treatment that enables local control of the affected region (17,22). However, it is unable to prevent relapse from other sites. The patient in the present study received post-operative radiation therapy, but relapsed after six months. An allogeneic bone marrow transplantation following induction therapy can reduce the risk of a subsequent systemic disease (11,23). Similar to the treatment of central nervous system leukemia, intrathecal injections may also be administered to patients with central nervous system myeloid sarcoma, particularly those with myeloblasts in the cerebrospinal fluid (23).

The present study described a case of myeloid sarcoma with primary intracranial lesions. The myeloid sarcoma recurred beside the lumbar vertebrae subsequent to surgery and radiation therapy. Although an ETO fusion gene was detected using fluorescence *in situ* hybridization, there was no evidence of leukemia based upon the morphological, immunophenotyping and chromosome band analyses of the bone marrow, which supports the hypothesis that the myeloid sarcoma occurred outside of the bone marrow. If the patient had received a bone marrow examination at the time of disease onset, an improved outcome may have been achieved following chemotherapy. As primary intracranial myeloid sarcoma is rare, further investigations are required in order to achieve an improved understanding of the mechanisms involved in the pathogenesis of myeloid sarcoma and to identify the neurophilic features that precede the onset of clinical leukemic disorders.
